# Contrast-enhanced MRI in evaluating the contralateral breast for synchronous malignancy in patients with invasive lobular cancer

**DOI:** 10.1186/bcr3284

**Published:** 2012-11-09

**Authors:** R Pietrosanu, MA Shah, H Gay, S Heller, M Reddy

**Affiliations:** 1 St Georges Hospital NHS Trust, London, UK

## Aim

To evaluate the use of MRI in assessment of the contralateral breast in women with invasive lobular cancer.

## Methods

Retrospective review of 157 breast MRIs in our local unit from January 2010 to May 2012 was carried out. Of these, 35 women had biopsy-proven invasive lobular cancer. Dotarem-enhanced 1.5T MRI was performed and UK standard reporting criteria were used to grade suspicion in any contralateral lesions alongside the use of contrast enhancement curves.

## Results

Bilateral MRI demonstrated nine (24%) contralateral lesions graded as MRI 2 or above, warranting further investigation with second-look ultrasound with five proceeding to ultrasound core biopsy. Of these, three were confirmed as malignant, revealing three contralateral invasive lobular cancers and one low-grade ductal carcinoma *in situ*. Our results demonstrated contralateral malignancy in 8% of women with invasive lobular disease.

## Conclusion

Current UK guidance does not routinely recommend MRI screening of the contralateral breast in patients with invasive lobular carcinoma. Studies have shown there is a wide variation in detection of contralateral malignancy from 3 to 18%. Our review showed a relatively high rate of incidental contralateral malignancy of 8%, suggesting that MRI screening of the contralateral breast may have more of a role in this patient group than initially considered. We are now extending this review to include three other hospitals in South West London Breast Cancer Network to evaluate these initial findings further.

